# Inhibitory activity of a standardized elderberry liquid extract against clinically-relevant human respiratory bacterial pathogens and influenza A and B viruses

**DOI:** 10.1186/1472-6882-11-16

**Published:** 2011-02-25

**Authors:** Christian Krawitz, Mobarak Abu Mraheil, Michael Stein, Can Imirzalioglu, Eugen Domann, Stephan Pleschka, Torsten Hain

**Affiliations:** 1 Institute for Medical Microbiology, Justus-Liebig-University, Frankfurter Strasse 107, 35392 Giessen, Germany; 2 Institute for Medical Virology, Justus-Liebig-University, Frankfurter Strasse 107, 35392 Giessen, Germany

## Abstract

**Background:**

Black elderberries (*Sambucus nigra *L.) are well known as supportive agents against common cold and influenza. It is further known that bacterial super-infection during an influenza virus (IV) infection can lead to severe pneumonia. We have analyzed a standardized elderberry extract (Rubini, BerryPharma AG) for its antimicrobial and antiviral activity using the microtitre broth micro-dilution assay against three Gram-positive bacteria and one Gram-negative bacteria responsible for infections of the upper respiratory tract, as well as cell culture experiments for two different strains of influenza virus.

**Methods:**

The antimicrobial activity of the elderberry extract was determined by bacterial growth experiments in liquid cultures using the extract at concentrations of 5%, 10%, 15% and 20%. The inhibitory effects were determined by plating the bacteria on agar plates. In addition, the inhibitory potential of the extract on the propagation of human pathogenic H5N1-type influenza A virus isolated from a patient and an influenza B virus strain was investigated using MTT and focus assays.

**Results:**

For the first time, it was shown that a standardized elderberry liquid extract possesses antimicrobial activity against both Gram-positive bacteria of *Streptococcus pyogenes *and group C and G *Streptococci*, and the Gram-negative bacterium *Branhamella catarrhalis *in liquid cultures. The liquid extract also displays an inhibitory effect on the propagation of human pathogenic influenza viruses.

**Conclusion:**

Rubini elderberry liquid extract is active against human pathogenic bacteria as well as influenza viruses. The activities shown suggest that additional and alternative approaches to combat infections might be provided by this natural product.

## Background

Elders (*Sambucus *spp.) are widely distributed throughout the world. In central Europe, the most common species are black elder (*Sambucus nigra *L.), red elder (*Sambucus racemosa *L.), and dwarf elder (*Sambucus ebulus *L.). Black elder is the most widespread, being found across Europe, central and western Asia, and northern Africa [1]. Black elder is a deciduous shrub that grows to a height of 4-6 m. From spring until summer the corymbs are in flower. The berries are dark violet-black drupes which grow in clusters and are only edible when fully ripe. Other parts of the plant, such as the green stems and branches, are not edible and not recommended for human consumption.

In 400 BCE, Hippocrates referred to the elder tree as his "medicine chest." Other noted classical healers, including Theophrastus, Dioscorides and Galen, regarded the elder as one of nature's greatest healing plants. The herbalist Hildegard von Bingen in the 12^th ^century, and the physician and author, Dr. Martin Blochwich in the 17^th ^century, continued to extol its virtues [2]. In the early 20^th ^century, British herbalist Maud Grieves provided a comprehensive review of the historical uses of black elder as a traditional medicine [3]. Prior to antibiotics, elderberry was found as one of the main ingredients in many preparations used by herbalists [4], pharmacists, and physicians. Today, elderberry is employed as an alternative to conventional medicines and mainly in the form of an extract for treating the common cold, influenza and Herpes virus infections [5-9]. Elderberry is often recommended for use as a complementary therapy together with the classic antioxidant nutrients, vitamin C and zinc, to support the natural process of recuperation [7].

The European black elderberries are rich dietary sources of plant pigments and phenolic compounds. They contain the flavonols, quercetin-3-glucoside and quercetin-3-rutinoside, and a number of anthocyanins: a group of phenolic compounds responsible for the attractive red, purple, and violet colours of many fruits, flowers, vegetables, and also elderberries. The anthocyanins of elderberries were identified as cyanidin-3-sambubioside-5-glucoside, cyanidin-3,5-diglucoside, cyanidin-3-sambubioside, cyanidin-3-glucoside, cyanidin-3-rutinoside, pelargonidin-3-glucoside, and pelargonidin-3-sambubioside. The anthocyanins of elderberries are bioactive; for example, able to enhance the postprandial plasma antioxidant status of healthy humans [10-13]. Animal and *in vitro *studies have shown that anthocyanins decrease necrotic and apoptotic cell death and lower infarct risks through anti-inflammatory and relaxant effects on coronary arteries [14].

Influenza virus (IV) infections cause seasonal epidemics and have the potential to become pandemic. Only a few medications are approved for use in the treatment of influenza A and B virus infections while they act directly and specifically against influenza viruses, the problem with these medications is that drug-resistance can develop relatively quickly [15,16]. Thus, there is an urgent need for new and more broadly based anti-influenza medications that do not allow resistance. Active substances with an unspecific inhibitory action against IV propagation--regardless of the viral subtype--would probably not lead to resistance, if such IVs do not evade inhibition by changes in their viral properties [17]. Moreover, bacterial super-infection during an ongoing IV infection can lead to severe pneumonia [18], and therefore substances with dual action against both types of pathogens--bacteria and IV--would be of further interest.

We have shown that a standardized elderberry liquid extract displays antimicrobial effects against the Gram-positive bacteria *Streptococcus pyogenes *and group C and G *Streptococci*, and the Gram-negative bacterium *Branhamella catarrhalis*, which often cause infections of the upper respiratory tract. As it was already known that elderberry extract can display activity against IV, we investigated the ability of a specific standardized extract of black elderberries to impair the propagation of human pathogenic influenza A and B virus strains, A/Thailand/KAN-1/2004 (KAN-1, H5N1) and B/Massachusetts/71 (Mass), in cell culture at non-toxic concentrations.

## Methods

### Rubini elderberry liquid extract

The extract used in the trials is a proprietary product known as Rubini and was supplied by BerryPharma AG (Solinger Strasse 7, D-42799 Leichlingen, Germany). This particular elderberry extract was chosen for our studies because it is standardized by HPLC and always produced from the same "Haschberg" variety of *S. nigra *L., which is grown under cultivation in the Steiermark region of Austria. The elderberry-to-extract ratio of the product is 18:1. The extract is concentrated and standardized using membrane filtration to achieve a minimum anthocyanin concentration of 3.2%. The concentration of anthocyanins is achieved using a mechanical filtration procedure in which semipermeable membranes separate substances according to their different molecular sizes. The HPLC assay is based on the IFU N° 71, 1998 method, measured at pH 1, 510 nm using cyanidin chloride (Sigma Aldrich) for the reference standard.

All references to elderberry liquid extract in this study refer to the same proprietary, standardized extract.

### Bacterial strains

Strains of *S. pyogenes*, group C and G *Streptococci*, and *B. catarrhalis *were directly isolated from patient samples and cultivated on sheep blood agar plates (37°C, 5% CO_2_) and refreshed twice-weekly. Patient isolates were characterized using different reference antibiotics (see Additional file 1, Table S1). For the experiments, bacteria were grown in an appropriate broth overnight at 37°C with shaking at 180 rpm (Unitron, Infors). Overnight cultures were diluted 1:50 in 20 ml fresh BHI (Brain Heart Infusion) broth using a 100-ml Erlenmeyer flask and were incubated at the conditions noted above until they reached an optical density of OD_600 nm _1.0.

### Cell line & viruses

Madin Darbin canine kidney cells (MDCK) were grown in DMEM (1x DMEM supplemented with 10% FCS, 100 U/ml penicillin, 100 μg/ml streptomycin). The following influenza virus strains were used: The human HPAIV isolate A/Thailand/KAN-1/2004 (KAN-1, H5N1) was supplied to S. Pleschka by P. Puthavathana, Thailand. The human strain B/Massachusetts/71 (B/Mass) was obtained from the IV strain collection in Giessen, Germany. KAN-1 and B/Mass were propagated on MDCK cells with low serum without trypsin for KAN-1 and with trypsin (2 μg/ml) for B/Mass. Strains were titrated by focus assay (see below).

### Testing of antimicrobial activity of elderberry liquid extract in liquid cultures

The strains were grown at 37°C at 180 rpm for 12-16 h. The optical density was measured at OD_600 nm _and differences were adjusted by taking different volumes to obtain the same amounts of bacteria.

Volumes of one milliliter fresh media were inoculated with 50 μl of bacteria overnight-cultured in 1.5-ml Eppendorf tubes. Elderberry liquid extract was added in amounts of 5%, 10%, 15%, or 20%. The prepared tubes were kept at 37°C and 180 rpm for another 16 h. Out of the tubes, 100 μl were diluted, plated on blood agar (Oxoid), and incubated at 37°C in broth for 24 h. Colony forming units (CFU) were counted and the counts were recalculated by factoring in the formerly made dilutions. Plated bacteria without any elderberry extract were set as 100% of possible growth. Growth figures from isolates exposed to elderberry liquid extract were set in relation to the strains not exposed to the extract. Every biological experiment was independently repeated at least three times with two replicates per trial.

### MTT assay

MDCK cells grown in 96-well microplates were incubated with DMEM/BA media (1x DMEM, 0.2% BA, 100 U ml^-1 ^penicillin and 0.1 mg ml^-1 ^streptomycin) with different concentrations of elderberry extract (as described under Materials and Methods, page 6) at 37°C, 5% CO_2 _for 12, 24, 36 and 48 h (16 wells per concentration and time point). The media was then aspirated and the cells were left to recover for 60 min in DMEM which was then replaced by 200 μl of MTT-mix (DMEM supplemented with 10% FCS and antibiotics containing 175 μg/ml MTT = 1-(4,5-dimethylthiazol-2-yl)-3,5-diphenylformazan; Sigma). The cells were further incubated for 90 min at 37°C and subsequently fixed with 4% PFA (in PBS) for 30 min at RT. The cells were dried and the tetrazolium crystals were dissolved by adding 200 μl of isopropanol to each well. The plates were shaken for 10 min and analyzed photometrically at 560 nm excitation in an enzyme-linked immunosorbent assay (ELISA) reader.

### Focus assay

MDCK cells grown in 96-well plates in DMEM to about 90% confluency were washed once with PBS++ and infected with 50 μl virus of a dilution resulting in about 100 foci/well in PBS/BA, for 1 h RT. The inoculum was aspirated and 150 μl MC-media (1x DMEM, 1.5% Methyl cellulose; Methocel MC, Fluka) for KAN-1 and Avicel-media (1x DMEM, 1.25% Avicel; FMC, Belgium) for B/Mass were added and the cells were incubated at 37°C, 5% CO_2_, for 36 h and 48 h. To detect foci of infected cells, the cells were fixed and permeabilized using 100 μl fixing solution (4% PFA, 1% Triton X-100 in PBS++) at 4°C for 60 min. The solution was then discarded and the cells were washed 3x with PBS++/0.05% Tween20 and further incubated with 50 μl 1st antibody (mouse-anti-Influenza A Nucleoprotein mAb, BIOZOL BZL 10908) diluted in PBS++, 3% BSA, for 1 h at RT. The cells were then were washed 3x with PBS++, 0.05% Tween20 and incubated with 50 μl of 2nd antibody (anti-mouse HRP-antibody) diluted in PBS++, 3% BSA, for 1 h at RT. The cells were then washed 3x with PBS++, 0.05% Tween20 and incubated in 40 μl/well "AEC" staining solution (Sigma) for 45 min at RT. After sufficient staining, the substrate was removed and the cells were washed 2x with dH_2_O to remove salts. To detect the size of foci (indicating a productive replication), the 96-well plates were scanned and analyzed using Photoshop software package.

### Statistical data analysis of experiments

All experiments were performed a minimum of three times. Significant differences between two values were compared with a paired Student's t-test. Values were considered significantly different when the *p *value was less than 0.05 (*p *< 0.05).

### Biosafety

All experiments using infectious virus were performed in accordance with German regulations applicable to the propagation of influenza viruses. All experiments involving highly pathogenic influenza A virus were performed at a biosafety level 3 (BSL3) containment laboratory approved for such use by the local authorities (RP, Giessen, Germany).

## Results

### Antimicrobial activity of elderberry liquid extract in bacterial liquid cultures

**Figure 1:**
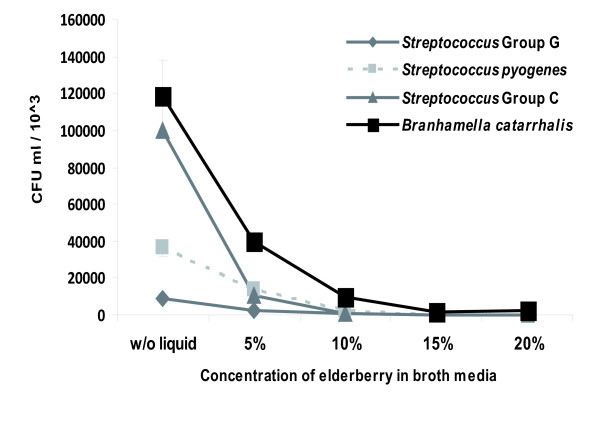
**Effect of the elderberry extract on bacterial growth**. Visualization of Gram-positive and Gram-negative bacterial strains exposed to different concentrations of Rubini elderberry extract during growth in liquid culture. Every biological experiment was independently repeated at least three times with two replicates per trial. Standard deviation is indicated. The *p *< 0.05 was observed for all bacterial species tested.

We used growth experiments in liquid cultures to assess the antimicrobial activity of a standardized elderberry liquid extract against Gram-positive bacteria of *S. pyogene*s and group C and G *Streptococci*, and the Gram-negative bacterium *B. catarrhalis*. CFUs were determined 16 hours after plating the bacteria on blood agar at different concentrations of the elderberry liquid extract (Figure 1). The addition of the elderberry extract at a concentration of 10% to bacterial strains in liquid culture media decreased their growth by >70% in comparison to untreated samples. A concentration of 20% elderberry liquid extract in broth media resulted in bacterial development under one percent of the originally measured values.

Antimicrobial activity of the elderberry liquid extract against the bacterial organisms in liquid broth was evident from lower concentrations of the extract compared to the results obtained with agar plates using disc diffusion methods. Whereas growth-inhibitory effects were found from the extract against *Haemophilus influenzae *using disc diffusion methods, it showed no inhibitory effects against the growth of the Gram-positive bacteria, *Staphylococcus aureus *(MRSA and MSSA) and *Streptococcus mutans*, and the Gram-negative bacterium of *H. parainfluenzae *(data not shown).

### Inhibitory activity of elderberry liquid extract on the propagation of B/Massachusetts/71 (B/Mass) and A/Thailand/KAN-1/2004 (KAN-1, H5N1) in cell culture by focus size reduction assay and titration of infectious particles

We first determined the concentration at which the extract could be tested for antiviral activity without cytotoxic effects by the MTT assay. This comprises the highest concentration of the extract that is still tolerable in cell culture and was expected to show the strongest activity and no, or barely any, cytotoxic activity. We found that a 1:100 dilution of the extract in media met both criteria.

MDCK cells were infected for 1 h with the IV strains B/Massachusetts/71 (B/Mass) and A/Thailand/KAN-1/2004 (KAN-1, H5N1) at a dose that allows easy focus detection. After removal of the inoculum, the cells were incubated for 48 h with overlay media in the presence of elderberry extract at the chosen concentration. The supernatant was then discarded and the foci size was determined by focus assay.

**Figure 2:**
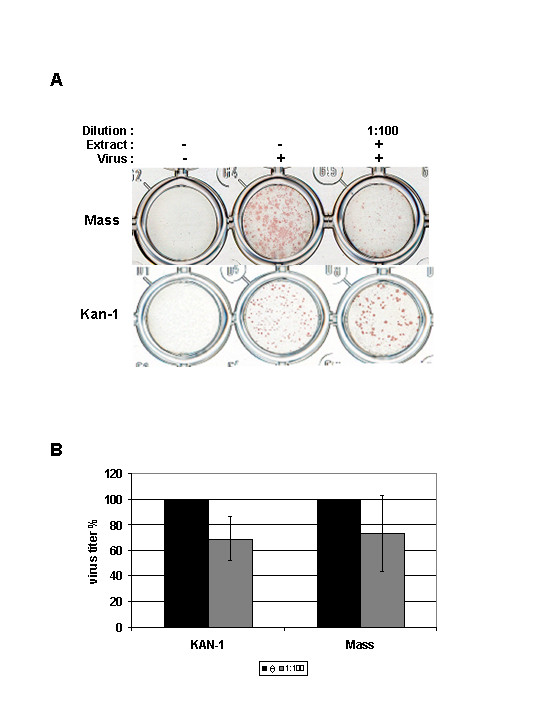
**Impact of the elderberry extract on propagation of IV**. **A) **Focus size reduction assay. MDCK cells were infected with the two virus strains as indicated and incubated for 48 h in the presence of the extract (1:100). Foci were detected by immunochemistry. **B) **Pre-treated MDCK cells were infected with pre-treated virus and were propagated for 48 h in the presence of the extract. As control untreated virus and cells were used for infection. The mean titre of three independent experiments is given as percentage of the control (black) set to 100%. Standard deviation is indicated (KAN-1: *p *< 0.07, Mass: *p *< 0.03).

At 48 h post-infection (p.i.), the elderberry extract treatment produced a clear reduction of foci size for B/Mass compared to the untreated control (Figure 2A). In summary, the elderberry liquid extract treatment reduces spread of the virus in cell culture. Interestingly, the results also show that the foci size of KAN-1 is actually not reduced but enlarged compared to the untreated control, whereas on the other side the result shows that the number of foci is reduced compared to the untreated control (Figure 2A). Furthermore, the total amount of virus produced after pre-treated cells were infected with pre-treated virus and incubated in the presence of the extract (1:100) was determined. For the respective virus strains, the results show that a reduction of about 30% (KAN-1) and 25% (Mass) can be achieved (Figure 2B).

## Discussion

The goal of this study was to determine the potential antimicrobial activity of a standardized elderberry liquid extract against several human bacterial and viral pathogens known to cause infections of the upper respiratory tract. We showed that the proliferation of *S. pyogenes*, group C and G *Streptococci*, and *B. catarrhalis *were reduced after contact with elderberry extract in liquid culture. Our results indicate that the applied concentrations of the elderberry liquid extract are an important antimicrobial parameter for further potential therapeutic treatment of bacterial pathogens.

The antimicrobial activity of an extract of elderberries (*S. nigra *L.) has been demonstrated against the growth of 13 common nosocomial Gram-positive and Gram-negative pathogens (e.g. *S. aureus *(MRSA), *Bacillus cereus, Escherichia coli *and *Pseudomonas aeruginosa*) using the disc diffusion technique [19]. Intriguingly, the authors reported that all noscomial strains, including *S. aureus *treated with an aqueous extract of the leaves at 10-fold dilution, failed to show any growth inhibitory activity, whereas 100-fold dilutions of freeze-dried, concentrates of ethanol extracts of the flowers or berries inhibited all the bacteria tested [19]. In our data, *S. aureus *(MRSA and MSSA) was not inhibited using Rubini elderberry liquid extract using disc diffusion assays. This strongly suggests that, compared to the elderberry extract prepared by membrane filtration used in our study, the extracts prepared by the method of Hearst and colleagues [19] may be significantly different. We speculate that the reasons for the different antimicrobial activities of the two elderberry extracts could depend on their chemical compositions or varying concentrations of antibacterial compounds within the extracts. Both studies demonstrate that elderberry extracts generated by different extraction methods may be useful as alternative or complementary medicines to potentially counteract the spread of certain kinds of bacteria responsible for upper respiratory tract infections. Finally, additional investigations are required to elucidate the mechanism of action of elderberry extracts against bacteria.

Syncytia inhibition assays have shown that elderberry extracts have strong antiviral activity against feline immunodeficiency virus (FIV) *in vitro *[20]. Also, flavonoids in the berries of *S. nigra *L. bind to and prevent H1N1-type IV infection *in vitro *[6]. Furthermore, influenza A and B virus bind to alpha2,3- and alpha2,6-linked sialic acid-linked glycoproteins as receptor determinants for infection via the viral haemagglutinin (HA) protein, and alpha2,6-linked sialic acid are recognized by the *S. nigra *L. agglutinin [21]. We therefore aimed to elucidate the anti-IV potential of the specific elderberry extract used in our investigations against a B-type IV (B/Mass) as well as a human isolate of a highly pathogenic avian IV (KAN-1, H5N1).

We have demonstrated that treatment of IV-infected cells with the elderberry liquid extract reduces B/Mass spread at concentrations that are non-toxic in cell culture and therefore should affect factors and/or mechanisms important for influenza B virus propagation. Interestingly, KAN-1 also showed a decrease in focus numbers, but at the same time, an enlargement in size. At the moment, this is not understood. Nevertheless, both virus strains show reduced titres after pre-treated MDCK cells were infected with the pre-treated strains, which were subsequently incubated in the presence of the extract for 48 h. The difference in the effect of the extract on the propagation of the two strains may reflect different viral dependencies on the factors or mechanisms blocked by the extract and therefore the extract might have different inhibitory potentials towards diverse types of IV. Looking at the different treatment regimens, pre-treatment of the cells had only a moderate effect in reducing KAN-1 titre in a single cycle replication (data not shown). The result would suggest that elderberry liquid extract blocks factors on the cell surface needed by KAN-1 (and therefore perhaps by other IV) for efficient infection of MDCK cells. It should be noted that the experiments were otherwise performed with pre-treated virus and cells and that the cells were further incubated in the presence of the diluted extract. Since preliminary results of other investigations indicate an inhibitory effect of elderberry liquid extracts against influenza A and B viruses [8,9], it can be concluded that the molecular target of elderberry extract is common to both viral genera. While this could be a specific as well as a nonspecific target, previous studies have shown that flavonoid components of the berries appear to exhibit a specific neuraminidase-inhibiting effect [4] and bind to the viral envelope of influenza [5]. Further work in elucidating the specific activity of Rubini elderberry liquid extract will allow a better understanding of its mode of action and therefore of its potential applications.

Regarding the usability of the elderberry liquid extract for *in vivo *testings, a compromise between concentration and low viscosity should be found to provide the best possible results. For practical purposes, an optimum route of administration would need to be determined and it would have to be decided which strategy to pursue in considering the potential of the extract. Combining the liquid extract with additional ingredients with the goal of increasing its effects might also be considered, just as it is possible that various natural ingredients could be used in combination with the extract for different kinds of therapeutic applications [22].

## Conclusion

Rubini elderberry liquid extract is active against human pathogenic bacteria as well as influenza viruses, both being clinically import groups of pathogens for which new and alternative therapeutic approaches are needed. In addition, bacterial super-infection during ongoing influenza virus infections complicates the situation for the patient. It would therefore be useful to simultaneously target both foes. The activities shown by the elderberry liquid extract suggest that additional and alternative approaches to influenza infections might be provided by natural products.

## Competing interests

The study was partially funded by BerryPharma AG (to S.P. and T.H.).

## Authors' contributions

MAM, ED, SP and TH designed the study. CK, MAM and MS carried out the laboratory studies. CK, MAM, SP, ED and TH analyzed the data and were involved in the preparation of the manuscript. CI collected the patient samples. All authors read and approved the final manuscript.

## Pre-publication history

The pre-publication history for this paper can be accessed here:

http://www.biomedcentral.com/1472-6882/11/16/prepub

## Supplementary Material

Additional file 1 **Antibiotic susceptibilities of the employed bacterial strains against penicillin G, doxycycline, clindamycin, cefazolin, cefuroxime and ceftazidime**. Table S1. Click here for file
