# Supplementary material for: Inhibitory activity of a standardized elderberry liquid extract against clinically-relevant human respiratory bacterial pathogens and influenza A and B viruses
Source: BMC Complement Altern Med. 2011 Feb 25;11:16. doi: 10.1186/1472-6882-11-16 (PMC3056848; doi:10.1186/1472-6882-11-16)
Supplement: Additional file 1 — Antibiotic susceptibilities of the employed bacterial strains against penicillin G, doxycycline, clindamycin, cefazolin, cefuroxime and ceftazidime. Table S1. [file 1472-6882-11-16-S1.PDF]

Supplemental Information

Table S1: Antibiotic susceptibilities of the employed bacterial strains against penicillin G, doxycycline, clindamycin, cefazolin, cefuroxime and ceftazidime.

**Bacterial strain**	**Susceptibilities against antibiotics (MIC in µg/ml)**					
	Penicillin G	Doxycycline	Clindamycin	Cefazolin	Cefuroxime	Ceftazidime
Streptococcus Group G	<0.064	>2.0	<0.5	<0.064	<0.064	<0.125
Staphylococcus aureus (MSSA)	>0.125	<1.0	<0.25	<4.0	<4.0	>256
Staphylococcus aureus (MRSA)	>256	<1.0	>256	>256	>256	>256
Streptococcus mutans	<0.064	>2.0	<0.5	<0.064	<0.064	<0.125
Streptococcus Group C	<0.064	<1.0	<0.5	<0.064	<0.064	<0.125
Streptococcus pyogenes	<0.064	<1.0	<0.5	<0.064	<0.064	<0.125
Haemophilus influenzae	1.0	<1.0	>4.0	>0.5	<1.0	<0.064
Haemophilus parainfluenzae	>1.0	<1.0	>4.0	>0.5	<1.0	<0.064
Branhamella catarrhalis	>1.0	<1.0	>4.0	>0.5	<1.0	<0.064

MIC = minimal inhibitory concentration
